# Social Functioning Profiles in Children with Autism Spectrum Disorder in China: A Preliminary Identification of Subtypes

**DOI:** 10.3390/bs16091616

**Published:** 2026-09-10

**Authors:** Xueyun Su, Jiajia Ge, Xiao Yang

**Affiliations:** 1 Department of Early Childhood Education, East China Normal University, Shanghai 200062, China; xysu@spe.ecnu.edu.cn; 2 China Research Institute of Care and Education of Infants and Young Children, Shanghai 200062, China; 3 Xiaoshan District Special Education School of Hangzhou, Hangzhou 311203, China; 51184107002@stu.ecnu.edu.cn

**Keywords:** autism spectrum disorder, social functioning, individual differences

## Abstract

Children with autism spectrum disorder (ASD) exhibit considerable heterogeneity in social functioning, suggesting the existence of distinct latent subtypes. A total of 479 Chinese children diagnosed with ASD (M_age_ = 9.338 years) were assessed using the Mandarin translation of the Stanford Social Dimensions Scale (SSDS). This measure aims to better comprehend individual differences in key social dimensions in ASD. Using the latent profile analysis (LPA) method, four profiles were identified and named based on their characteristics, and differences among the profiles were validated using data from the Full-scale Intelligence Quotient (FSIQ), Social Communication Questionnaire (SCQ), Strengths and Difficulties Questionnaire (SDQ), and Social Responsiveness Scale-Second Edition (SRS-2). The results revealed that (1) the social functioning level of children with ASD can be divided into four profiles: Risky, Apathetic, Active but Awkward, and Advantageous. (2) Children in the Advantageous profile showed fewer ASD-related symptoms and more prosocial behaviors than those in the other profiles. This study offers preliminary evidence for the heterogeneity of social functioning in children with ASD, serving as a step toward precision-based social interventions and inclusive educational support.

## 1. Introduction

Autism spectrum disorder (ASD) is a heterogeneous neurodevelopmental disorder characterized by persistent deficits in social communication and social interaction across multiple contexts, as well as restricted and repetitive patterns of behavior, interests, or activities (American Psychiatric Association, 2022). Impairment in social functioning is one of the core symptoms of children with ASD. Research has increasingly advanced our understanding of the multidimensional and complex nature of social functioning. Previous studies strongly support the emphasis on social motivation, social communication, and social cognition as central components of social processing. Social motivation refers to the behaviors and predispositions that drive people to seek social interactions and to form and maintain social bonds, with its components supported by the brain’s reward system (Pudlo et al., 2022). Social communication refers to the appropriate understanding and use of verbal and nonverbal communication within a social context (Jethava et al., 2022). Social cognition is a crucial transdiagnostic construct with clinical and functional relevance across a range of neuropsychiatric disorders and encompasses social knowledge, perception and processing of social cues, and the representation of mental states (Fortier et al., 2016; Lakhani et al., 2021). Persistent difficulties with social cognition often make it hard for individuals with ASD to respond in ways that conform to social norms (Vuattoux et al., 2021). Although impairments in social functioning are common among children with ASD, there are substantial individual differences in its severity and developmental course (Fong & Iarocci, 2020). Research has shown that individual differences in social cognition and social communication can help identify potential subtypes in individuals with ASD based on the compensation framework (Livingston et al., 2019). Such individual differences in social functioning also imply that intervention needs and appropriate methods may differ among children with ASD (Beglinger & Smith, 2005). Meanwhile, it has been suggested that improvement in a single social competence domain (e.g., social cognition) does not necessarily translate into enhanced overall social functioning (Turner-Brown et al., 2008). Existing research collectively demonstrates the significant clinical and research value of subtyping social functioning in children with ASD: a single, categorical approach cannot adequately account for the significant heterogeneity among individuals, nor can it provide precise guidance for personalized interventions.

However, existing studies on social functioning subtypes in children with ASD still have several limitations. Firstly, social functioning is inherently context-dependent, and whether a behavior is considered socially appropriate is shaped, in part, by the social norms and expectations of a particular cultural context. According to related cross-cultural research, cultural expectations and cultural value orientation may influence the social deficits of people with ASD (Cheng et al., 2025; Freeth et al., 2013). For example, a study examined the extent of stigma faced by individuals with ASD and noted that Eastern collectivist culture emphasizes interpersonal harmony and indirect communication, whereas Western individualistic culture places greater emphasis on self-expression and direct interaction (Cheng et al., 2025). At present, most research on the social functioning subtypes of ASD is mainly conducted in Western or other specific regional samples, and there is a lack of empirical testing of whether the classification framework can be directly applied to children with ASD in the Chinese cultural context. These limitations mean that, in the Chinese cultural context, exploratory research on potential types of social functioning in children with ASD using reliable standardized instruments is of urgent theoretical and practical significance. On the one hand, identifying localized subtypes helps to accurately describe the characteristics of social functional heterogeneity in children with ASD in China. On the other hand, different subtypes may correspond to different intervention needs and appropriate approaches. Existing interventions for social functioning in children with ASD, such as the Early Start Denver Model, Pivotal Response Treatment, and Reciprocal Imitation Training, have demonstrated beneficial effects. Their effectiveness may vary across different dimensions of social functioning. Similarly, existing assessment instruments also weaken practical application due to reasons such as the lack of a specific, comprehensive assessment of social motivation or the limited number of entries measuring social motivation while capturing lighter symptom expression (Phillips et al., 2019). For example, a widely used social dysfunction assessment instrument, the Social Communication Questionnaire (SCQ), does not cover the assessment dimension of social recognition ability (Chandler et al., 2007).

To address the limitations of existing instruments for measuring social motivation, Jennifer M. Phillips and colleagues developed the Stanford Social Dimensions Scale (SSDS) based on extensive previous research (Phillips et al., 2019). This instrument measures five dimensions: social motivation (SM), social affiliation (SA), expressive social communication (ESC), social recognition (SR), and unusual approach (UA). Its strengths lie in its comprehensiveness, multidimensionality, and strong psychometric properties. Although existing research has identified distinct social functioning profiles in children with ASD using the SSDS (Uljarevic et al., 2020), whether these profiles are applicable across cultural contexts remains unclear. Social functioning is embedded within culturally shaped norms and values, and macro-level social, economic, and cultural changes may substantially influence socialization goals and practices and the development of social competence (Chen & French, 2008). Consistent with this perspective, previous cross-cultural studies have reported differences in the expression of social and ASD-related behaviors across countries (Matson et al., 2011). Therefore, examining social functioning profiles in children with ASD in China may help identify potentially culture-specific patterns of social functioning. Such findings may also provide a preliminary basis for developing more culturally responsive and individualized intervention strategies for children with ASD.

This study aimed to examine the heterogeneity of social functioning among children with ASD in the Chinese context using the Mandarin version of the SSDS, which has demonstrated adequate reliability and validity in China (Ge et al., 2026). Specifically, this study first used the Mandarin version of the SSDS to explore whether children with ASD could be classified into distinct profiles based on their social functioning, and to describe the specific social functioning patterns of any identified profiles. Then, to further characterize these profiles, we compared them in terms of IQ, ASD-related symptoms, gender, and age.

## 2. Methods

### 2.1. Ethics

This study was approved by the Ethics Committee of East China Normal University (Ethics Approval ID: HR-025-2021). Throughout this study, the researchers strictly adhered to the guidelines and procedures established by the ethics committee. During participant recruitment, the study’s purpose, content, and procedures were clearly explained to all participants. All individuals involved in the study participated voluntarily and could withdraw at any time without incurring any liability.

### 2.2. Participants

This study employed a convenience sampling method. Participants were eligible if their parents reported that their child had received a clinical diagnosis of ASD from a qualified clinician in a medical institution in China, following established diagnostic procedures. To facilitate the recruitment of children with clinically diagnosed ASD, recruitment posters were distributed online to parents through special schools, regular schools, and educational intervention institutions across different regions of China. The online questionnaire was preceded by a participant information statement outlining the research purpose, instructions for completing the questionnaire, and contact information. The sample consisted of 405 boys (84.551%) and 74 girls (15.449%), with a mean age of 9.338 years (range = 2–17 years). Participants were distributed across five age groups: 2–5 years (*n* = 68), 6–8 years (*n* = 136), 9–11 years (*n* = 135), 12–14 years (*n* = 97), and 15–17 years (*n* = 43).

In addition, as an incentive for participation, parents received individualized reports on their children’s social functioning, along with corresponding recommendations, after completing the SSDS questionnaire. SCQ, SDQ, and SRS-2 data were also collected as supplementary and sample-characterization information. FSIQ scores were available for 110 participants based on previous hospital-administered assessments, as reported by their parents. The specific Wechsler assessment instrument varied according to the child’s age at the time of assessment. Participant characteristics are summarized in Table 1.

### 2.3. Instrumentation

#### 2.3.1. Stanford Social Dimensions Scale (Mandarin Version)

The SSDS contains a total of 71 items, including five dimensions: Social Motivation, Social Affiliation, Expressive Social Communication, Social Recognition, and Unusual Approach. Exploratory factor analysis revealed that the scale has good construct validity, demonstrating an adequate fit ([CFI] = 0.940, [TLI] = 0.919, [RMSEA] = 0.048, [SRMR] = 0.038) (Phillips et al., 2019). The results of administering the SSDS in Mandarin Chinese within the context of Chinese culture indicate that its factor structure is reproducible, with excellent internal consistency reliability, composite reliability, and retest reliability. There is ample evidence of both convergent validity and discriminant validity (Ge et al., 2026). Therefore, this scale is suitable for measuring the social functioning of children with ASD in China.

#### 2.3.2. Strengths and Difficulties Questionnaire (Parent-Reported Version)

The SDQ (Goodman et al., 1998) consists of 25 items organized into five dimensions: emotional symptoms, conduct problems, hyperactivity–inattention, peer problems, and prosocial behavior. The total difficulties score is calculated by summing the first four dimensions, while the fifth dimension assesses the children’s positive attributes. The prosocial score is not reversed and added to the total difficulties score, as lacking prosocial behaviors is conceptually different from having psychological difficulties. Items are rated on a three-point Likert scale ranging from “not true” to “certainly true”. Both the parent-report and self-report versions of the SDQ scale demonstrate good psychometric properties (Muris et al., 2003). Nevertheless, given concerns about the accuracy of self-reports from children with ASD, the parent-report version was selected for this study.

#### 2.3.3. Social Responsiveness Scale-Second Edition (SRS-2)

The SRS-2 (Constantino & Gruber, 2012) is a widely used measurement designed to assess the severity of autism-related symptoms and quantify social impairment (Turi et al., 2025). It can be completed by parents, teachers, or other caregivers within 15–20 min. The scale comprises 65 items organized into five dimensions: social awareness, social cognition, social communication, social motivation, and restricted interests and repetitive behavior. The first four dimensions form the Social Communication and Interaction (SCI) subscale, while the last constitutes the Restricted Interests and Repetitive Behavior (RRB) subscale. Items are rated on a four-point Likert scale, and the total score is obtained by summing all item responses, ranging from 0 to 195. After standardization, the raw total score is converted into a standard score (*T*-score) to determine the level of symptom severity, with higher scores indicating more severe social impairment. Specifically, *T*-scores of 76 or above indicate severe impairment, 66–75 indicate moderate impairment, 60–65 indicate mild impairment, and *T*-scores of 59 and below indicate the normal range (Bruni et al., 2014).

#### 2.3.4. Social Communication Questionnaire (SCQ)

The Social Communication Questionnaire (SCQ), developed by Rutter and Lord, is a parent-report screening scale based on the Autism Diagnostic Interview–Revised (ADI-R), including three dimensions: reciprocal interaction (RI, 15 items), communication (C, 13 items), and restricted, repetitive and stereotyped patterns of behavior (RRSB, 8 items). The SCQ has a recommended cutoff score of 15, with higher scores indicating greater levels of social and communication difficulties (Berument et al., 1999). Exploratory factor analysis of the Mandarin translation of the SCQ identified a three-factor structure comprising social interaction, repetitive behaviors, and communication. This structure was further validated by confirmatory factor analysis, which demonstrated an adequate fit ([GFI] = 0.923, [CFI] = 0.983, [RMSEA] = 0.034) (Gau et al., 2011).

### 2.4. Data Processing and Analysis

Latent profile analysis (LPA) was conducted using Mplus 8.0 to identify distinct profiles of children with ASD based on their SSDS scores across five social functioning subscales. A one-way ANOVA was conducted using SPSS 27.0 to compare the identified profiles on the five SSDS subscale scores. To further characterize the profiles, Pearson’s chi-square test was used to examine differences in gender, while one-way ANOVA or Welch’s ANOVA was used, as appropriate, to examine differences in IQ, age, and ASD-related symptoms (measured by the SCQ, SDQ, and SRS-2) across the profiles.

## 3. Results

### 3.1. Potential Profile Analysis of the Social Functioning for Children with ASD in China

To identify potential profiles of social functioning in children with ASD, this study used LPA on the items of the Stanford Social Dimensions Scale (SSDS). Referring to the recommendations of existing studies (Huang et al., 2021; Marsh et al., 2009; Wang et al., 2025), the optimal number of social functioning profiles was determined based on the following criteria: (1) the Akaike information criterion (AIC), Bayesian Information Criterion (BIC), and Adjusted Bayesian Information Criterion (ABIC) were considered, with lower values indicating better relative model fit; (2) entropy, ranging from 0 to 1, with values closer to 1 indicating greater classification accuracy, and values above 0.70 generally considered indicative of adequate classification quality; (3) the Lo–Mendell–Rubin likelihood ratio test (LMR-LRT), for which a significant result (*p* < 0.05) indicates that the k-profile model provides a better fit than the k−1-profile model; (4) the Bootstrap likelihood ratio test (BLRT), for which a significant result (*p* < 0.05) indicates that the k-profile model provides a better fit than the k−1-profile model; and (5) the proportion of participants in each profile, with each profile generally expected to include at least 5% of the sample. The final profile solution was selected by considering the overall pattern of model fit, classification quality, profile size, parsimony, and substantive interpretability rather than relying on any single criterion.

A series of models with 1–8-profile solutions were estimated, and the model fit indices are shown in Table 2. Entropy values exceeded 0.70 across all multi-profile solutions, indicating high classification accuracy. As the number of profiles increased, the BLRT remained significant (*p* < 0.001). Although the LMR-LRT was not significant for the comparison between three- and four-profile solutions (*p* = 0.187), the four-profile solution showed a substantial reduction in the information criteria (ΔAIC = 1227.523, ΔBIC = 981.393, and ΔABIC = 1168.653). Importantly, the four-profile solution also provided a more differentiated and substantively interpretable representation of the multidimensional patterns of social functioning. In contrast, the addition of a fifth profile yielded a comparatively smaller incremental improvement in model fit. The LMR-LRT remained nonsignificant (*p* = 0.787), while the reductions in the information criteria were considerably smaller than those observed when moving from three to four profiles (ΔAIC = 334.883, ΔBIC = 88.753, and ΔABIC = 276.012), and entropy increased only marginally from 0.962 to 0.966, suggesting little improvement in classification quality. Although the six-profile solution continued to improve the information criteria, it increased model complexity without providing sufficient additional improvement in model quality or substantive interpretability. Consequently, considering the overall pattern of fit indices, classification quality, profile size, parsimony, and substantive interpretability, a four-profile model was selected as the preferred solution.

### 3.2. Identification and Characterization of Social Functioning Profiles for Children with ASD

The results of the one-way ANOVA and Welch’s ANOVA showed significant differences across the four profiles on all five SSDS subscales: SM (*F* = 254.917, *p* < 0.001, Partial *η*^2^ = 0.644), SA (*F* = 307.214, *p* < 0.001, Partial *η*^2^ = 0.660), ESC (*F* = 273.118, *p* < 0.001, Partial *η*^2^ = 0.633), SR (*F* = 207.950, *p* < 0.001, Partial *η*^2^ = 0.555) and UA (*F* = 6.844, *p* < 0.001, Partial *η*^2^ = 0.071). Based on the scores of the four potential profiles of social functioning for children with ASD on the five SSDS subscales, the profiles were labeled according to their distinctive characteristics (see Table 3). The labels were intended as concise descriptive shorthand to facilitate the interpretation and communication of the distinct social functioning patterns identified in the latent profile analysis. They were developed through multiple rounds of discussion among the researchers, considering the correspondence between each label and the salient characteristics of the corresponding profile. Importantly, these labels do not represent diagnostic categories or fixed ASD subtypes, nor are they intended to capture the full range of characteristics of the children within each profile. Rather, they describe the relative pattern of social functioning observed in the present sample.

Profile 1 had the lowest scores on four of the five subscales (SM, SA, ESC, and SR) but the highest score on UA, indicating pronounced difficulties across overall social functioning and was thus named the Risky profile, accounting for the lowest percentage (11.1%). Profile 2 showed a generally moderate pattern of social functioning, with the lowest UA score among the four profiles. It was therefore named the Apathetic profile, accounting for 33.4% of the total sample. Profile 3, which had the highest percentage (40.5%), had the second-highest scores on SM, SA, ESC, and SR among the four profiles, indicating relatively strong functioning across multiple social dimensions. It was thus named the Active but Awkward profile. Profile 4 had the highest scores across SM, SA, ESC, and SR, indicating the strongest functioning across the four social functioning dimensions. It was therefore named the Advantageous profile, accounting for 15.0% of the total sample.

### 3.3. Differences Among the Four Profiles in IQ, ASD-Related Symptoms, and Demographic Characteristics

To examine differences among the identified social functioning subtypes in Full- scale Intelligence Quotient (FSIQ), ASD-related symptoms, gender, and age, we conducted a series of Pearson’s chi-square, one-way ANOVA, Welch’s ANOVA, and post hoc tests using SPSS 27.0 (see Table 4). Among the 110 participants with available FSIQ data, the overall mean FSIQ was 63.155 (SD = 28.685), with 9, 40, 42, and 19 participants contributing FSIQ data in Profiles 1–4, respectively. Welch’s ANOVA indicated significant differences in FSIQ across the four profiles (*F* = 12.588, *p* < 0.001, Partial *η*^2^ = 0.262). Post hoc comparisons indicated that children in Profile 4 (the Advantageous profile) had the highest FSIQ scores, whereas Profile 1 (the Risky profile) had the lowest scores.

Significant differences were observed among the four profiles in SCQ. For social interaction, Welch’s ANOVA indicated significant profile differences with a large effect size (*F* = 159.348, *p* < 0.001, *η*^2^ = 0.477). Post hoc comparisons showed that Profile 1 exhibited the highest social interaction difficulties, followed by Profiles 2 (the Apathetic profile) and 3 (the Active but Awkward profile), whereas Profile 4 showed the lowest difficulties. For the communication domain, one-way ANOVA revealed significant differences among profiles (*F* = 46.617, *p* < 0.001, *η*^2^ = 0.227). Profile 4 showed significantly lower communication difficulties than Profiles 1, 2, and 3, while Profile 3 showed lower scores than Profiles 1 and 2. For repetitive behaviors, Welch’s ANOVA also revealed significant differences, although with a relatively small effect size (F = 8.499, *p* < 0.001, *η*^2^ = 0.045). Profile 2 showed higher repetitive behavior scores than Profiles 3 and 4.

Concerning child psychopathology measured by the SDQ, significant differences were identified in peer problems (*F* = 7.262, *p* < 0.001, Partial *η*^2^ = 0.044). Children in Profile 4 showed higher levels of peer problems compared with those in Profile 1. Significant differences were also found for prosocial behavior (*F* = 86.290, *p* < 0.001, Partial *η*^2^ = 0.373), with Profile 4 demonstrating the highest prosocial behavior scores, whereas Profiles 1 and 2 showed relatively lower levels of prosocial behavior.

Regarding ASD-related symptoms measured by the SRS-2, significant differences were observed in social communication and interaction difficulties (*F* = 80.084, *p* < 0.001, Partial *η*^2^ = 0.327). Children in Profile 4 exhibited the lowest levels of social communication and interaction, whereas those in Profile 2 demonstrated the highest levels. Significant differences were also found for restricted interests and repetitive behaviors (*F* = 7.871, *p* < 0.001, Partial *η*^2^ = 0.052). Profiles 2 and 3 showed relatively elevated levels of restricted and repetitive behaviors compared with Profile 4. The four profiles did not differ significantly in age, as indicated by a one-way ANOVA (*F* = 1.325, *p* = 0.265, Partial *η*^2^ = 0.008), or in gender distribution, as indicated by Pearson’s chi-square test (χ^2^ = 1.611, *p* = 0.657, Cramer’s V = 0.058).

## 4. Discussion

Children with ASD exhibit significant heterogeneity in their social functioning. Using the Mandarin version of the SSDS, this study explored potential profiles of social functioning in children with ASD. The four subtypes were then compared on demographic characteristics and ASD-related symptoms to further clarify each subtype’s characteristics.

This study employed latent profile analysis to investigate the latent subtypes of social functioning among children with ASD in China. The results identified four distinct subtypes of social functioning: Risky, Apathetic, Active but Awkward, and Advantageous. The four profiles were distinguished by different patterns of relative strengths and weaknesses across the five SSDS subscales. The Advantageous profile showed the highest scores on SM, SA, ESC, and SR, whereas the Risky profile showed the lowest scores on these four subscales. The Apathetic and Active but Awkward profiles fell between these two extremes in terms of overall performance, with distinct relative differences across the SSDS subscales. Furthermore, the profiles identified by this study showed no differences in age or gender, but exhibited a significant gradient in FSIQ across different social functioning subtypes.

When findings from the SCQ, SDQ, and SRS-2 were considered alongside the profile characteristics, an interesting finding was that children in the Advantageous profile, despite demonstrating relatively strong social functioning and prosocial behaviors, still exhibited elevated peer relationship problems. Previous research indicates that children with ASD may participate in social interactions and express a desire for friendship while still struggling to establish reciprocal peer relationships (Kasari et al., 2011). While social skills training is widely used, previous research has suggested that interventions aimed at reducing social communication differences between children with ASD and typically developing children may actually harm peer friendships among children with ASD (Granieri et al., 2020). One possible interpretation is that some children with ASD may develop compensatory or socially learned interaction strategies that facilitate observable social participation but may not necessarily translate into deeper interpersonal reciprocity. In addition, peer relationship difficulties may partly reflect mutual misunderstandings between children with ASD and non-ASD peers, consistent with the “double empathy problem” perspective (Milton, 2012). However, these interpretations remain tentative in the present study, as measures of compensatory behaviors, interaction styles, or friendship quality were not included. These possibilities highlight the importance of considering not only observable social skills but also the quality of reciprocity of social relationships when understanding social functioning in children with ASD.

### 4.1. Cross-Cultural Understanding and Caregiver Empowerment for Social Functioning Subtypes

A comparison of the evidence on Chinese subtypes from this study with that from previous research (Uljarevic et al., 2020) reveals that, although both studies used the SSDS to identify heterogeneity in the social functioning of children with ASD, four subtypes were identified in the Chinese sample, whereas Uljarevic et al.’s sample (which included multiple ethnicities, such as Caucasian, Asian, and mixed-race) yielded five profiles (moderate but expressive communication and affiliation impaired; socially severe; moderate but SR impaired; mild; adaptive). The most significant differences were observed in the distribution of UA: in the profile with the best social functioning across both studies (SM, SA, ESC, and SR scores), profile 5 (labeled as Adaptive) in Uljarevic et al.’s study had the highest UA scores. In contrast, the Advantageous profile in this study showed the highest scores on SM, SA, ESC, and SR but a relatively lower UA score. The UA items capture behaviors perceived as unusual in terms of their intensity and content, including social initiations and approaches revolving around one’s unusual or intense interests and routines (Phillips et al., 2019). These findings suggest that stronger overall social functioning does not necessarily coincide with the same relative pattern across all SSDS subscales. Cultural context may partly contribute to the observed discrepancy, as culturally shaped expectations regarding socially appropriate interaction may influence how unusual approaches are perceived. However, this interpretation should be treated with caution, as the observed difference cannot be attributed to cultural factors alone and may also reflect differences in sample characteristics, clinical contexts, and measurement conditions.

As noted in the introduction, social functioning is influenced by cultural expectations regarding appropriate interpersonal behavior, communication styles, and peer interactions. The meaning and expression of different social functioning profiles may therefore vary across cultural contexts. Accordingly, the identification and interpretation of social functioning subtypes among children with ASD should take the local cultural context into account, including culturally specific social norms, educational expectations, and patterns of interpersonal interaction. Children with ASD are also influenced by multiple levels of their environments in the development of social functioning, such as social cognition. Therefore, there is a growing need for culturally adaptive interventions for individuals with ASD (Davenport et al., 2018). One possible contextual factor that may contribute to such cultural variation is the family environment. Parents of children with ASD often face a significant caregiving burden, including a decreased quality of life, impacts on work and social life, and the development of psychological disorders (Cheatham & Fernando, 2022). In the Chinese context, social stigma may represent an additional source of stress for caregivers. Research indicates that caregivers of children with ASD in China frequently experience a sense of alienation due to social stigma (Zheng et al., 2024). Stigma may further influence parents’ perceptions of their children’s social development and the strategies they use to support it. For example, some parents may hope that their children will behave in ways that are more aligned with mainstream social norms and become “normal” in order to avoid social exclusion. In an effort to manage stigma, some caregivers either refrain from social contact or conceal their child’s condition, consequently restricting the child’s access to social involvement and developmental progress (Ng & Ng, 2022).

Caregivers play an important role in the decisions and the implementation of social functioning interventions for children with ASD. Identifying distinct social functioning profiles may provide caregivers and practitioners with information about children’s specific areas of difficulty and thereby inform the consideration of individualized support strategies. For example, for children with the Risky profile who score lowest on expressive social communication (ESC), augmentative and alternative communication (AAC) may be considered as one potential support for facilitating functional communication and social participation. Previous research has suggested that AAC interventions can support children with ASD to use a broader range of communicative functions, including social-communication functions (Logan et al., 2017). However, the appropriateness and effectiveness of AAC may vary across individuals and should be considered in relation to each child’s communication needs and response to intervention.

### 4.2. Implications for ASD Subtyping and Social Functioning Interventions

The heterogeneity of individuals with ASD highlights the importance of identifying meaningful subgroups to provide a basis for targeted interventions. Consequently, there is a growing consensus in the research community about the need to integrate appropriate methods for precise subtype identification. Existing studies have explored subtype identification from various perspectives; for example, some have adopted a neuroimaging approach by clustering large-scale functional brain connectivity profiles (Easson et al., 2019). Other studies, based on a resting-state fMRI dataset comprising 940 individuals with ASD, identified two subtypes: a low-connectivity subtype and a high-connectivity subtype, with the high-connectivity subtype being associated with relatively higher severity of social-emotional symptoms (Pagani et al., 2026). Other studies have identified subtypes based on genetic and phenotypic data. For example, a large-scale study in 2025 incorporated phenotypic information from 5392 children with autism (covering 239 distinct trait items) and employed a general finite mixture model (GFMM) for analysis. It ultimately identified four subtypes with fundamental differences at the behavioral, genetic, molecular, and cellular levels: Moderate Challenges (34%), Broadly Affected (10%), Social/Behavioral (37%), Mixed ASD with DD (19%) (Litman et al., 2025). Similarly, Kang and colleagues categorized children with ASD into four subtypes based on 13 atypical communication characteristics: Speech Delay + Pragmatic Difficulty + Fixated Language, Pervasive Atypical Communication Characteristics, Little Professors, and Moderate Pragmatic Difficulty Only (Kang et al., 2020). The heterogeneity of social functioning, as a key source of variation within the core symptom domain of ASD, influences the social performance and intervention needs of individuals across different subtypes. Therefore, identifying distinct subtypes of social functioning has become a key approach to better understanding the heterogeneity of individuals with ASD and implementing personalized interventions.

Although the results from different measures indicate a possible association between social functioning profiles and ASD-related characteristics, the specific profile patterns are not entirely consistent. This study found that children in the Advantageous profile exhibited the lowest levels of social communication and interaction difficulties and restricted/repetitive behaviors on the SRS-2, while children in the Apathetic profile showed the highest levels on both SRS-2 subscales. Children in the Risky profile, which was characterized by relatively poorer overall social functioning, showed the highest levels of difficulties in social interaction and communication on the SCQ, although their repetitive behavior scores were not the highest. Thus, these findings are consistent with the pattern that social functioning profiles were characterized by heterogeneous patterns of strengths and weaknesses across social components rather than a simple severity gradient (Uljarevic et al., 2020). Previous research has indicated that core autism symptoms are associated with reduced social motivation (Pallathra et al., 2018) and diminished social pleasure (Carré et al., 2015), which may reduce children’s willingness to initiate or maintain social interactions. This decline in willingness may be further reflected in a decrease in the frequency of actual social participation. Children with greater impairment in social and recreational skills tend to participate less frequently in social activities, thereby reducing opportunities for social learning and reciprocal peer engagement (Orsmond et al., 2004). Restricted and repetitive behaviors may further pose challenges to children’s social participation. For example, behavioral rigidity, repetitive patterns of speech or action, and highly restricted interests may reduce behavioral flexibility and make it more difficult for children with ASD to adapt to dynamic social situations with different interaction partners. Together, difficulties in social communication and interaction and restricted and repetitive behaviors may contribute to challenges in reciprocal peer interactions and participation in social environments. However, these potential mechanisms were not directly examined in the present study and should therefore be interpreted as possible explanations for the observed profile differences rather than causal pathways.

Although all participants of this study met diagnostic criteria for ASD, children demonstrated markedly different patterns of social functioning, supporting the prevailing view that ASD diagnosis alone may not adequately capture individual differences in social adaptation and participation. Importantly, the social functioning profiles identified in the present study should not necessarily be interpreted as fixed or immutable categories. Increasing evidence suggests that ASD-related characteristics and social interaction patterns may change across development. For example, longitudinal research has shown that social interaction styles in children with ASD may shift over time, with some individuals becoming more socially active or socially typical as autism symptoms decrease (Scheeren et al., 2020). Similarly, a systematic review demonstrated substantial longitudinal heterogeneity in autistic trait trajectories, including increasing, decreasing, and stable developmental patterns (Pender et al., 2020). Even the autism diagnostic status itself may show developmental changes in some individuals (Elias & Lord, 2022). Taken together, these findings suggest that social functioning profiles may represent dynamic patterns of functioning at a given developmental stage rather than stable or fixed categories. Although challenges in social communication and restrictive/repetitive behaviors may persist throughout an individual’s life (Rahman & Rawitt, 2026), targeted interventions that address individual strengths and difficulties across social functioning domains may, to some extent, facilitate social adaptation for individuals with ASD.

### 4.3. Limitations and Future Research

Several limitations should be acknowledged. In this study, the dimensional analysis of the SSDS and the examination of specific differences in social functioning among children with ASD were largely based on data-driven results, which may deviate from real-world conditions. Given that there are differences in the descriptions provided by various reporting sources, the manifestations of social functioning in children with ASD may vary (Junttila et al., 2006). Future studies should incorporate multiple sources of information and contextual observations.

FSIQ data were available for only 110 of the 479 participants, with relatively small and unequal numbers across the four profiles. Therefore, the between-profile differences in FSIQ should be interpreted cautiously, particularly for profiles with fewer available cases. Future studies should recruit larger samples with more complete and systematically collected cognitive assessment data to further examine the relationship between cognitive ability and social functioning profiles.

Furthermore, the subtypes identified in this study were based on parent-reported data, which are subject to potential informant bias (Scheeren et al., 2020). Although we attempted to enhance the reliability of parent-reported assessments by recruiting participants through channels that broadly reached the ASD parent community and by providing parents with relevant assessment reports to facilitate their evaluation, parent reports may not fully capture individual differences in symptom manifestation across different contexts. In addition, because recruitment and questionnaire completion were conducted online, families with limited Internet access or less familiarity with electronic questionnaires may have been less likely to participate and may therefore have been under-represented in the sample. Therefore, contextual factors should be taken into account when making judgments (Lerner et al., 2017). Since this study is based on behaviors of social functioning in children with ASD, there may be some discrepancies compared to subtypes identified through biological markers or neuroimaging related to pathophysiology.

In short, these findings should therefore be considered preliminary; future validation should integrate multisource assessment, neuroimaging, and molecular genetic subtyping to achieve more precise subtype characterization.

## 5. Conclusions

This study used latent profile analysis to identify four distinct social functioning profiles among children with ASD in China based on the Mandarin version of the Stanford Social Dimensions Scale (SSDS): Risky, Apathetic, Active but Awkward, and Advantageous. These profiles differed significantly in FSIQ and several measures of ASD-related symptoms, but not in gender or age. Our findings provide empirical evidence for meaningful heterogeneity in social functioning among children with ASD, highlighting the need for individualized, profile-based interventions. Future research should combine data-driven results with theoretical frameworks, multi-informant input, and contextual assessments to further validate the subtype structure and enhance ecological validity.

## Figures and Tables

**Table 1:** Participant characteristics (*n* = 479).

	M (SD) or *n* (%)		M (SD)
Age (years)	9.338 (4.287)	SRS-2 Total *T*-score	66.679 (4.711)
Male	405 (84.551)	SCQ Total score	20.221 (6.926)
Female	74 (15.449)	SDQ Total difficulties score	34.008 (5.115)
FSIQ^#^	63.155 (28.685)	SDQ Prosocial behavior score	8.127 (2.394)

Notes. FSIQ^#^ = Full-scale Intelligence Quotient (*n* = 110); SCQ = Social Communication Questionnaire; SDQ = Strengths and Difficulties Questionnaire; SRS-2 = Social Responsiveness Scale-Second Edition.

**Table 2:** Model fit indices for the latent profile analysis.

Model	AIC	BIC	ABIC	Entropy	BLRT	LMR-LRT	Smallest Group (%)
1 Profile	79,815.714	80,299.632	79,931.461	-	-	-	1.00
2 Profiles	73,885.422	74,615.469	74,060.039	0.961	<0.001	<0.001	49.50
3 Profiles	71,905.182	72,881.360	72,138.671	0.954	<0.001	0.504	20.00
4 Profiles	70,677.659	71,899.967	70,970.018	0.962	<0.001	0.187	11.10
5 Profiles	70,342.776	71,811.214	70,694.006	0.966	<0.001	0.787	6.70
6 Profiles	69,546.419	71,260.988	69,956.521	0.964	<0.001	0.757	5.40
7 Profiles	69,086.574	71,047.273	69,555.546	0.959	<0.001	0.739	3.80
8 Profiles	68,782.421	70,989.250	69,310.264	0.956	<0.001	0.760	3.80

Notes. AIC = Akaike Information Criterion; BIC = Bayesian Information Criterion; ABIC = Adjusted Bayesian Information Criterion; BLRT = Bootstrap Likelihood Ratio Test; LMR-LRT = Lo–Mendell–Rubin Likelihood Ratio Test.

**Table 3:** Profile comparisons across SSDS subscales.

	Profile 1 Mean (SD)	Profile 2 Mean (SD)	Profile 3 Mean (SD)	Profile 4 Mean (SD)	Statistics	Post Hoc
SM	27.491 (8.217)	27.638 (5.039)	38.510 (5.632)	50.889 (7.557)	*F* = 254.917, *p* < 0.001, *η*^2^ = 0.644	1, 2 < 3 < 4
SA	13.887 (3.688)	20.163 (3.482)	25.155 (3.388)	31.444 (4.083)	*F* = 307.214, *p* < 0.001, *η*^2^ = 0.660	1 < 2 < 3 < 4
ESC	12.943 (3.134)	19.350 (2.923)	22.433 (2.748)	27.583 (3.668)	*F* = 273.118, *p* < 0.001, *η*^2^ = 0.633	1 < 2 < 3 < 4
SR	10.453 (3.016)	15.925 (3.769)	20.041 (3.144)	24.222 (4.088)	*F* = 207.950, *p* < 0.001, *η*^2^ = 0.555	1 < 2 < 3 < 4
UA	13.151 (4.339)	10.494 (2.808)	11.294 (2.344)	11.014 (2.464)	*F* = 6.844, *p* < 0.001, *η*^2^ = 0.071	1 > 3 > 2; 1 > 4

Notes: SM = social motivation; SA = social affiliation; ESC = expressive social communication; SR = social recognition; UA = unusual approach; 1/2/3/4 = profile 1/2/3/4.

**Table 4:** Alignment of other dimensions of ASD-related symptoms and demographics on the social functioning latent profiles.

	Dimension	Profile 1 M (SD)	Profile 2 M (SD)	Profile 3 M (SD)	Profile 4 M (SD)	Statistics	Post Hoc
FSIQ	-	41.000 (12.679)	50.950 (18.644)	68.095 (25.005)	88.421 (37.772)	*F* = 12.588, *p* < 0.001, *η*^2^ = 0.262	1, 2 < 3 < 4
SCQ	Social interaction	11.774 (3.023)	9.894 (2.404)	6.294 (3.111)	3.361 (2.519)	*F* = 159.348, *p* < 0.001, *η*^2^ = 0.477	4 < 3 < 2 < 1
	Communication	7.925 (2.311)	7.625 (2.276)	5.959 (2.078)	4.528 (1.921)	*F* = 46.617, *p* < 0.001, *η*^2^ = 0.227	4 < 3 < 1, 2
	Repetitive behaviors	4.453 (2.569)	5.263 (2.191)	4.536 (2.598)	3.681 (2.318)	*F* = 8.499, *p* < 0.001, *η*^2^ = 0.045	4, 3 < 2
SDQ	Emotional symptoms	8.208 (2.692)	8.463 (1.815)	8.119 (1.852)	8.083 (1.867)	*F* = 1.240, *p* = 0.297, *η*^2^ = 0.007	NS
	Conduct problems	7.377 (2.078)	7.388 (1.160)	7.613 (1.461)	7.472 (1.39)	*F* = 0.914, *p* = 0.436, *η*^2^ = 0.005	NS
	Hyperactivity–inattention	9.057 (2.349)	9.506 (1.542)	9.263 (1.641)	9.250 (1.734)	*F* = 1.157, *p* = 0.326, *η*^2^ = 0.007	NS
	Peer problems	8.245 (2.174)	8.831 (1.437)	9.052 (1.502)	9.528 (1.592)	*F* = 7.262, *p* < 0.001, *η*^2^ = 0.044	1 < 2, 3 < 4
	Total difficulties score	32.887 (7.780)	34.188 (4.158)	34.046 (5.089)	34.333 (4.642)	*F* = 0.516, *p* = 0.672, *η*^2^ = 0.006	NS
	Prosocial behavior score	6.434 (2.398)	6.813 (1.534)	8.660 (1.904)	10.861 (2.203)	*F* = 86.290, *p* < 0.001, *η*^2^ = 0.373	1, 2 < 3 < 4
SRS-2	Social communication and interaction	62.755 (6.201)	64.875 (2.889)	61.546 (2.726)	57.417 (4.103)	*F* = 80.084, *p* < 0.001, *η*^2^ = 0.327	4 < 1,3 < 2
	Restricted interest and repetitive behavior	77.698 (11.519)	81.206 (7.239)	79.686 (9.267)	74.875 (10.453)	*F* = 7.871, *p* < 0.001, *η*^2^ = 0.052	4 < 2; 4 < 3
CA	-	9.138 (4.265)	9.746 (4.579)	9.348 (4.270)	8.555 (3.590)	*F* = 1.325, *p* = 0.265, *η*^2^ = 0.008	NS
Gender	-	-	-	-	-	χ^2^ = 1.611, *p* = 0.657, Cramer’s V = 0.058	NS

Notes: FSIQ = Full-scale Intelligence Quotient; SCQ = Social Communication Questionnaire; SDQ = Strengths and Difficulties Questionnaire; SRS-2 = Social Responsiveness Scale-Second Edition; CA = Chronological age; NS = Not significant; 1/2/3/4 = Profile 1/2/3/4.

## Data Availability

Data are contained within the article.

## Abbreviations

The following abbreviations are used in this manuscript:

Children with ASD	Children with autism spectrum disorder
SCQ	Social Communication Questionnaire
SDQ	Strengths and Difficulties Questionnaire
SRS-2	Social Responsiveness Scale-Second Edition
SSDS	Stanford Social Dimensions Scale
